# Anti‐fat attitudes among Spanish general population: Psychometric properties of the anti‐fat attitudes scale

**DOI:** 10.1111/cob.12543

**Published:** 2022-07-18

**Authors:** Sergio Macho, Ana Andrés, Carmina Saldaña

**Affiliations:** ^1^ Departament de Psicologia Clínica i Psicobiologia Universitat de Barcelona (UB), Passeig Vall d'Hebron 171, 08035 Barcelona Spain; ^2^ Faculty of Psychology, Education and Sport Sciences Ramon Llull University Barcelona Spain; ^3^ Institut de Neurociències Universitat de Barcelona (UB) Barcelona Spain

**Keywords:** AFA Spanish adaptation, confirmatory factor analysis, exploratory factor analysis, test–retest reliability, weight discrimination

## Abstract

Weight discrimination is one of the worst forms of prejudice and is deeply rooted in society. The aim of this study was to adapt the anti‐fat attitudes scale (AFA) to the Spanish general population. The sample consisted of 1248 participants from the Spanish community population. They were recruited through the internet and participated voluntarily. Women (77.8%) were more predominant than men. Regarding body weight categories, 5.3% were underweight, 43.5% were normal‐weight, 24.9% were overweight and 26.3% had obesity. A cross‐validation method with an exploratory and confirmatory factor analysis confirmed the three‐factor structure of the AFA. The Spanish version of the AFA showed a satisfactory internal consistency for all three factors, as well as adequate test–retest reliability after a 1‐month interval. Finally, the Spanish version of the AFA seems to be an adequate tool to assess negative attitudes towards obesity in both clinical and research settings. Men presented more negative attitudes towards obesity and were convinced that obesity is under someone's control. Women presented more fear of gaining weight. Normal‐weight people were those who discriminated more. Participants with overweight or obesity suffered more fear of gaining weight. There was no intra‐group discrimination between individuals with overweight and obesity.

**WHAT IS ALREADY KNOWN ABOUT THIS SUBJECT**

- Weight discrimination is one of the most common and harmful types of discrimination.
- Negative attitudes towards obesity are the basis of weight discrimination.

**WHAT THIS STUDY ADDS**

- The adaptation of the anti‐fat attitudes scale (AFA) to the Spanish general population.
- A valid scale (AFA) for assessing anti‐fat attitudes in Spanish populations, including participants among all BMI categories.

## 1 INTRODUCTION

Obesity is a chronic health condition present worldwide that is considered a pandemic by the World Health Organization^1^ due to its epidemiology. In 2016, 13% of the world's adult population had obesity, reaching rates of 44% in some countries, e.g. in the United States.^2^ Obesity is more present in women than men^3^ and is more common than underweight.^4^

Obesity is related to a wide variety of medical^5^, ^6^ and psychological conditions, such as depression and anxiety.^7^, ^8^ Obesity is also linked to weight discrimination, one of the worst forms of stigmatization. This is based on the bias of weight. Weight bias is related to non‐reasonable judgements about people's weight.^9^ These judgements are commonly directed towards people with obesity as people with higher body weights are those who suffer more weight discrimination.^10^, ^11^ They are usually considered as lazy, lacking in self‐discipline or having poor willpower.^12^ Negative attitudes or beliefs towards obesity are also present in children and teenagers.^13^, ^14^

This kind of discrimination is strongly accepted in society.^15^ As a result, it is present across a wide variety of life areas such as employment, health and educational settings.^16^ Mass media also plays an important role in the perpetuation of the negative stereotypes about obesity, whereby male characters play these negative stereotypes more often than women.^17^, ^18^ Bias towards obesity is also present in a wide variety of professions, especially in healthcare professionals such as pharmacists,^19^ nurses,^20^ primary care professionals^21^ and also in exercise and nutrition professionals.^22^, ^23^ The same is also found in professionals treating eating disorders.^24^ These attitudes can be a detriment in the healthcare of people with obesity and can also damage the patient professional relation.

Weight stigmatization has also been associated with a wide range of health outcomes. In particular, depression, anxiety, low self‐esteem or body image dissatisfaction are common consequences of experiencing weight stigma.^25^, ^26^ Other health outcomes, like medication non‐adherence or substance abuse have also been associated with weight discrimination.^27^

Previous studies indicate that weight stigmatization is particularly attributed to unhealthy eating and exercise behaviours.^28^ Facing weight discrimination increases food intake and binge eating behaviours.^29^, ^30^ At the same time, it also promotes the avoidance of physical exercise.^31^, ^32^ These behaviours exacerbate the weight‐gain cycle and maintain or increase the probability of being on the receiving end of weight discrimination.

There is a classical differentiation of types of attitudes when studying discrimination: implicit and explicit attitudes. The main differences between them are in the automaticity and controllability of each attitude. Implicit attitudes are automatic and out of control, while explicit ones are intentional and can be controlled.^33^ A wide range of methods and scales have been used to assess these negative attitudes towards obesity. In 2017, a systematic review^34^ provided a summary of the psychometric properties of self‐reported questionnaires to assess weight bias. The study compiled 40 self‐report questionnaires and their psychometric properties. Despite the wide range available, the most popular and most used scales are the attitudes towards obese persons scale (ATOP),^35^ the beliefs about obese persons scale (BAOP)^35^ and the anti‐fat attitudes scale (AFA).^36^ To date, the AFA has been adapted to Spanish speakers in a sample of normal weight college students,^37^ but there is a lack evidence of the psychometric properties of the scale using a community sample of adults with a wide range of BMI.

The main aim of the study was to analyse the psychometric properties of the AFA in its Spanish version applied to a wide community sample including all BMI categories. Specifically, the aims were to: (1) study the internal structure of the scale using a principal component analysis (PCA) and a confirmatory factor analysis (CFA); (2) analyse the reliability of the scale in terms of internal consistency and test–retest and (3) analyse the possible differences in weight bias among sex, BMI and other sociodemographic variables.

We expected to find the three factors structure of the AFA original scale and the previous Spanish validation of the scale. At the same time, we also expected to find adequate stability and internal structure values. It was hypothesized that, as previous literature stated: (1) men will reveal more negative attitudes towards obesity than women, (2) women will provide higher values of fear of gaining weight, (3) participants with overweight and obesity will also feel dislike towards other individuals with obesity.

## 2 METHOD

### 2.1 Participants

Initially, 1423 participants were enrolled in the study. However, 175 did not meet the inclusion criteria (to be 18 years or older, to report their weight and height and to have completed the AFA scale). Consequently, the final sample comprised 1248 participants.

Women were the most predominant (77.8%) and men represented 22.2% of the total sample. The mean age was 33.36 years (SD = 10.57, range from 18 to 73 years). The mean BMI was 26.81 kg/m^2^ (SD = 6.85, range from 11.72 to 65.91 kg/m^2^). Regarding BMI categories, 5.3% participants were underweight, 43.5% were normal weight, 24.9% were overweight and 26.3% participants had obesity. Regarding income, we established different cut‐off points based on the Spanish minimum interprofessional wage.

### 2.2 Measures

#### 2.2.1 Sociodemographic questionnaire

An ‘ad hoc’ questionnaire was created to assess the main sociodemographic variables (e.g., sex, age and income range). Participants also reported their weight and height. Subsequently, BMI was calculated and classified into BMI categories according to the World Health Organization^1^ classification.

#### 2.2.2 Anti‐fat attitudes scale

The AFA^36^ is a multi‐factorial questionnaire formed by 13 items. The first factor is a 7‐item factor called *Dislike* and includes feelings about people with overweight and/or obesity. The second factor is a 3‐item factor called *Fear of Fat*, which measures the fear of gaining weight. Finally, the third factor is a 3‐item factor called *Willpower*. This last factor measures the extent to which participants believe obesity is under someone's control. Higher scores indicate higher anti‐fat attitudes in each factor. This Spanish validation of the AFA was conducted using a sample of college students without a representation of participants with overweight and/or obesity. This was the main motivation to repeat the validation using a large sample that came from the general population and that included a representation of participants from all the BMI categories. In the present study, the Spanish version of the AFA was applied,^37^ but the Likert scale was also changed using a 9‐point Likert scale (from ‘1’ completely disagree to ‘9’ completely agree), according to the original version of the questionnaire.^36^

#### 2.2.3 Multi‐dimensional scale of perceived discrimination

The multi‐dimensional scale of perceived discrimination (MSPD)^37^ is a 20‐item questionnaire developed to measure perceived discrimination. This is not a specific scale to assess perceived weight discrimination. It can be used to assess any type of discrimination, as items can be adapted (e.g., race or sexual discrimination). The MSPD presents four subscales: blatant group discrimination (BGD), subtle group discrimination (SGD), blatant individual discrimination (BID) and subtle individual discrimination (SID). It is one of the few instruments to differentiate both implicit and explicit weight bias attitudes. However, in this study, participants only responded to the BGD and SGD scales, because the others were more appropriate for assessing individuals that had perceived weight discrimination. Cronbach's alpha indicates good coefficients for each subscale (BGD = 0.88, SGD = 0.79). A 5‐point Likert scale from 1 = strongly disagree to 5 = strongly agree was used to assess the scale. Higher scores indicated lower discrimination. In our study, internal consistency of the MSPD was assessed in both subsamples. Cronbach's alpha for the BGD subscale ranged from 0.881 to 0.865 and McDonald's omega ranged from 0.885 to 0.869. For the SGD subscale, Cronbach's alpha ranged from 0.823 to 0.796 and McDonald's omega ranged from 0.827 to 0.797.

### 2.3 Procedure

The study was posted in different social networks. We wanted to benefit from the snowball effect of such networks in order to recruit the maximum number of participants. No company was paid to spread or advertise the study. Data was collected via a secure internet‐based platform. Participants were asked to participate voluntarily, and no compensation was given. The study was described as a body image study to reduce social desirability, which could have affected the results if it was described as a weight discrimination study.

Participants first had to read the essential information and give their informed consent. Confidentiality of their personal information was guaranteed. The study was previously verified and approved by the Bioethics Committee of the University of Barcelona.

Participants were also required to be involved in a second phase of the study. Participation was also voluntarily and no reward was given. Those participants who decided to participate in this second part and provided their email address were contacted after 1 month (+3 to 6 days), in order to assess the test–retest reliability of the scale. Anonymity was also guaranteed in this second phase, as participants were identified with an alphanumerical code. Thus, we were able to pair up the two measures taking during the study while respecting the participants' anonymity.

A total of 202 participants participated in the second part of the study 1 month later: 161 women (79.7%) and 41 men (20.3%). This gender ratio was similar to the gender ratio of the original sample. The mean age was 35.54 years (SD = 9.78) and the mean BMI was 26.58 kg/m^2^ (SD = 5.92).

### 2.4 Statistical analysis

All statistical analyses were performed using IBM SPSS 24 version (IBM Corp., Armonk, NY) and IBM SPSS Amos Statistics 20 version (IBM Corp., Chicago, IL). There were no missing data, as all participants who did not complete the questionnaire in full were removed before the analysis. This criterion was also applied to the test–retest sample.

First, a cross‐validation procedure was conducted.^38^ This method consisted of randomly dividing the sample into two independent samples. Subsample 1 (*n* = 624) was used to study the PCA and subsample 2 (*n* = 624) was used to conduct the CFA. Both subsamples were equivalent, as Student's *t*‐test and chi‐squared test showed no differences in terms of mean age (*p* > .05), mean BMI (*p* > .05) or sex ratio (*p* > .05). The reliability analysis and analysis of the relationships between the AFA scale and other variables were performed using the entire sample (*n* = 1248).

Second, a descriptive analysis of the main variables and a PCA were carried out. To conduct the PCA, the Kaiser–Meyer–Olkin (KMO) test and Barlett's sphericity test were chosen to analyse the adequacy of this statistical test. PCA following Varimax rotation was used. Factors were retained if eigenvalues were greater than 1.0,^39^ and also according to parallel analysis^40^ and the scree‐test results.^41^

Third, a CFA was performed using IBM SPSS Amos Statistics, version 20. Maximum Likelihood method was selected. The goodness‐of‐fit indices that were selected were chi‐square test, goodness‐of‐fit index (GFI), formed fit index (NFI), comparative fit index (CFI), Tucker–Lewis index (TLI), the standardized root mean square residual (SRMR) and the root mean square error of approximation (RMSEA). To determine the goodness of fit of the CFA, we chose a cut‐off equal to or greater than 0.90 for GFI and NFI,^42^ a cut‐off value close to 0.95 for CFI and TLI,^43^ a cut‐off value close to 0.08 for SRMR,^43^ and a cut‐off value close to 0.06 for RMSEA.^43^

Fourth, reliability was studied using Cronbach's alpha and McDonald's omega test using the entire sample (*n* = 1248) and interpreted according to Bland and Altman.^44^ Stability was also analysed based on the test–retest reliability using Pearson's correlation and interpreted according to Akoglu's^45^ recommendations.

Finally, to study the relationship between AFA subscales and other variables, analysis of variance (ANOVA), Student's *t*‐test and Pearson's correlation test were conducted. ANOVA was used to study the relation between AFA subscales and BMI categories. Games‐Howell and Hochberg GT2 post hoc comparisons were applied. Student's *t*‐test was performed to study the relationship between AFA subscales among sex. ANOVA and Student's *t*‐test were complemented with Hedges' *g* with a 95% confidence interval (IC) and interpreted following the Cohen^46^ criteria. Pearson's correlation was used to study the relationship between AFA subscales and the subscales of the MSPD. These correlations were interpreted according to Akoglu^45^ values.

## 3 RESULTS

### 3.1 Sociodemographic characteristics

The sociodemographic characteristics of the participants are represented in Table 1. The mean age was 33.36 years (SD = 10.57). The vast majority of the participants were normal weight (43.5%), followed by participants with obesity (26.3%). The mean BMI was 26.81 kg/m^2^ (SD = 6.85). As for sex, 971 were women (77.8%) and 277 were men (22.2%). Education level was divided into three categories: primary, secondary and higher studies. More than half of the sample had completed higher education (66.5%). Only 0.7% participants had only completed primary education. A total of 39.7% of the sample had an income less than the minimum wage and 25.2% earned less than 2 times the minimum wage. There were also participants that reported receiving no income (students or unemployed participants). Almost all of the participants were Caucasian or European (92.4%). Other ethnic groups were in the minority.

**TABLE 1:** Participants' sociodemographic information (*n* = 1248)

Variables	*n* (%)
Sex	
Female	971 (77.8%)
Male	277 (22.2%)
BMI category	
Underweight	66 (5.3%)
Normal weight	543 (43.5%)
Overweight	311 (24.9%)
Obesity	328 (26.3%)
Education	
Primary	9 (0.7%)
Secondary	409 (32.8%)
Higher education	830 (66.5%)
Income	
Any income	300 (24%)
Minimum wage	495 (39,7%)
2 minimum wage	315 (25.2%)
2.5 minimum wage	138 (11.1%)
Race	
White (Caucasian and European)	1153 (92.4%)
Latin	59 (4.7%)
Black	4 (0.3%)
Combined	32 (2.6%)

### 3.2 Principal component analysis

The PCA was conducted using subsample 1 (*n* = 624). Principal Axis Factoring extraction with Varimax rotation was conducted. The Kayser–Meyer–Olkin test (KMO = 0.816) and Bartlett's Sphericity test (*χ* ^2^_[78]_ = 3489.128; *p* < .001) revealed the adequacy of the data. The results showed that the scale followed a three‐factor solution, explaining the 33.04%, 17.11% and 13.63% of the variance for each factor. The scree‐plot and parallel analysis also suggested that three factors should be retained. As shown in Table 2, the factor *Dislike* (*Antipatía* in Spanish) had 7 items, while the other two dimensions (*Miedo a la ganancia de peso* and *Falta de voluntad* in Spanish) had three items each of them. All factor loadings were considered acceptable.

**TABLE 2:** Factor loadings of the AFA in subsample 1 (*n* = 624)

Item	Description^a^	Communalities	Factor 1	Factor 2	Factor 3
1	Dislike overweight people	0.512	0.622		
2	Not having overweight friends	0.164	0.349		
3	Overweight people are unreliable	0.549	0.740		
4	Overweight people are less intelligent	0.646	0.803		
5	Not taking seriously an overweight person	0.727	0.849		
6	Felt uncomfortable with overweight people	0.605	0.752		
7	Overweight people should not be employed	0.512	0.628		
8	Feeling bad about gaining weight	0.818		0.895	
9	Gain weight is one of the worst things	0.791		0.881	
10	Worried about being fat	0.785		0.884	
11	Overweight people could lose some weight doing exercise	0.669			0.812
12	Overweight people have lack of willpower	0.800			0.883
13	Overweight people are responsible of their weight	0.712			0.807
	Eigenvalue		4.295	2.223	1.772

^a^ These are not the items of the scale. The original Spanish translation can be consulted in Magallares and Morales.^47^

### 3.3 Confirmatory factor analysis

To confirm the structure of the AFA, a CFA was performed. In this analysis, data from subsample 2 (*n* = 624) was used. Goodness‐of‐fit indices indicated that the data fitted the proposed three‐factor model: *χ* ^2^_(62)_ = 302.397, *p* < .01, CFI = 0.933, TLI = 0.915, GFI = 0.921, NFI = 0.917, RMSEA = 0.079 (95% CI: 0.070 to 0.088), SRMR = 0.059. All values were adequate, according to the established cut‐off values.

**FIGURE 1:**
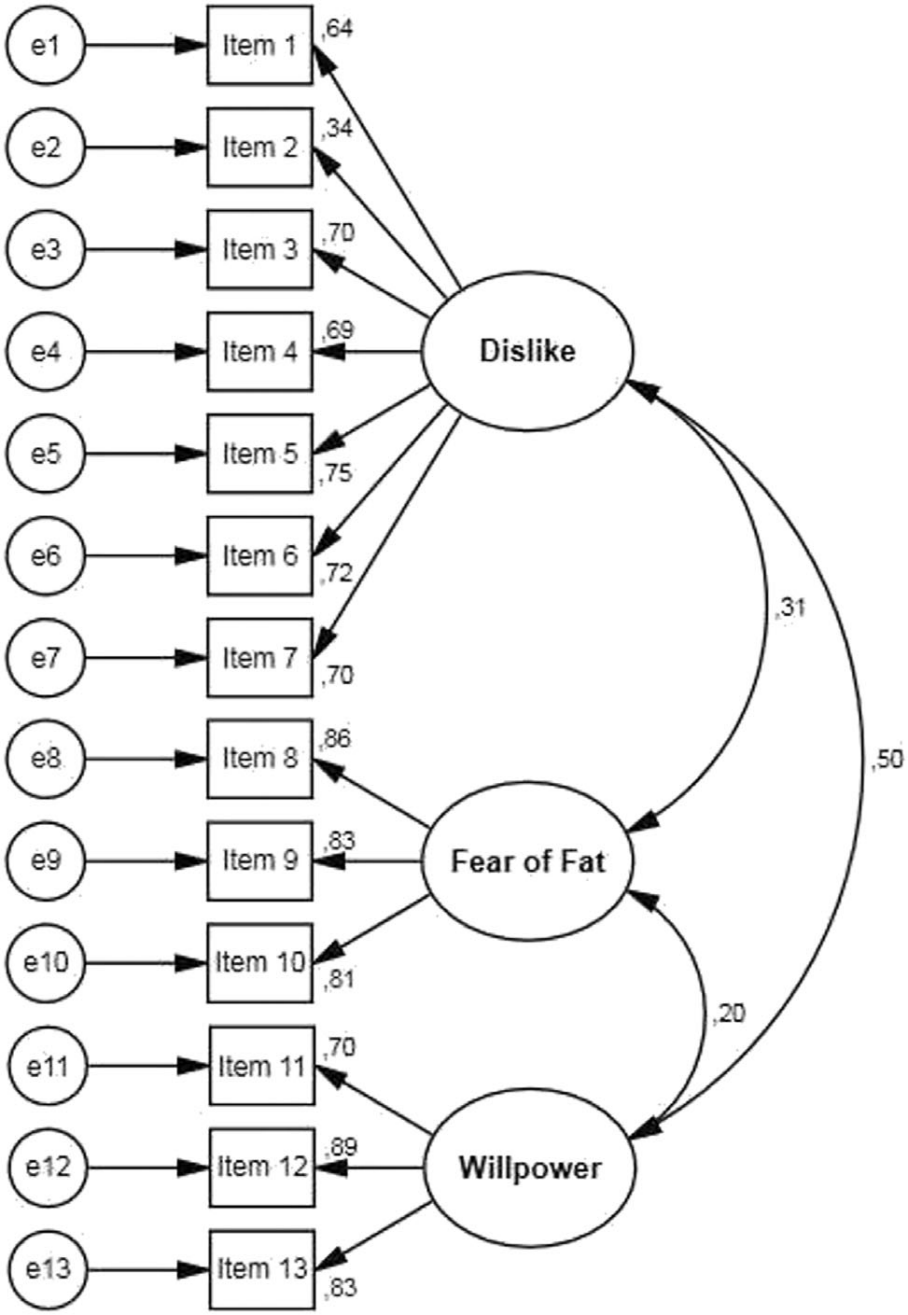
Confirmatory factor analysis path diagram for the Spanish version of the anti‐fat attitudes scale

The CFA confirmed the multi‐dimensional structure of the scale, formed by three factors. Standardized regression weights were acceptable in all items, ranging from 0.34 to 0.89 (Figure 1).

### 3.4 Internal consistency and test–retest reliability

The scale's internal consistency was evaluated using Cronbach's alpha and McDonald's omega in both subsamples (Table 3). All these results show excellent reliability values. There were no overwhelming differences if an item was removed. Therefore, all items were finally kept. Furthermore, item‐total correlations were adequate in both subsamples, as shown in Table 3.

**TABLE 3:** Internal consistency analysis of the AFA in both subsamples

		Sample 1 (*n* = 624)				Sample 2 (*n* = 624)			
	Number of items	α	ω	I‐T corr (range)	I‐T corr (mean)	α	ω	I‐T corr (range)	I‐T corr (mean)
Dislike	7	0.789	0.836	0.329–0.661	0.559	0.793	0.839	0.325–0.659	0.566
Fear of fat	3	0.874	0.875	0.740–0.784	0.759	0.872	0.872	0.739–0.774	0.755
Willpower	3	0.819	0.829	0.629–0.752	0.680	0.841	0.850	0.646–0.772	0.712

The scale was administered again 1 month later to the 202 participants who agreed to participate in the second part of the study, in order to determine the test–retest reliability of the scale. Test–retest analysis suggested that the scores were strongly associated even after a month (+3 to 6 days). Pearson's correlation analysis between the two parts of the study showed *r* = .690 (*p* < .001) for *Dislike*; *r* = .723 (*p* < .001) for *Fear of Fat* and *r* = .680 (*p* < .001) for *Willpower*. All correlations were strong or close to strong correlations.

### 3.5 Convergent validity

Table 4 shows correlations between AFA and other related variables used to assess the convergent validity of the scale. The table shows a significant correlation between the *Dislike* subscale from AFA and the explicit discrimination (BGD) subscale from the MSPD. However, no statistically significant correlation was found between the *Dislike* subscale and implicit discrimination (SGD). It should be noted that higher scores on the BGD and SGD subscales from the MSPD indicate lower perceived discrimination levels. There were also significant correlations between the *Willpower* dimension from the AFA, implicit (SGD) and explicit discrimination (BGD). These results highlight the conviction that obesity is something controllable and related to more implicit and explicit perceived group discrimination.

**TABLE 4:** Convergent validity of the AFA

		AFA scale			MSPD scale	
		1	2	3	4	5
AFA scale	(1) Dislike	–				
	(2) Fear of fat	0.260***	–			
	(3) Willpower	0.403***	0.128***	–		
MSPD scale	(4) BGD	−0.107***	0.195***	−0.334***	–	
	(5) SGD	0.036	0.205***	−0.201***	0.720***	–

Abbreviations: BGD, blatant group discrimination; SGD, subtle group discrimination.

* *p* < .05; **p < .01; ***p < .001.

### 3.6 Relationship between the AFA and other sociodemographic variables

Table 5 shows the mean and standard deviation for all the scales of the AFA for the different BMI categories. As shown, there were significant differences in the three factors of the scale among the BMI categories. For *Dislike* towards obesity (*F* = 8.213, df = 3, *p* < .01), normal weight people presented more negative attitudes and beliefs towards obesity than overweight people (*p* < .05, Hedges' *g* = 0.21, 95% CI = 0.07 to 0.35) and people with obesity (*p* < .001, Hedges' *g* = 0.32, 95% CI = 0.19 to 0.46).

**TABLE 5:** ANOVA comparisons of the AFA among BMI categories

	UW mean (SD)	NW mean (SD)	OW mean (SD)	O mean (SD)	ANOVA	Post hoc comparisons	Hedges' *g* (95% CI)
Dislike	2.12 (1.20)	2.43 (1.41)	2.15 (1.16)	2.01 (1.08)	*F*_(3)_ = 8.213	NW > OW*	0.21 (0.07 to 0.35)
						NW > O***	0.32 (0.19 to 0.46)
Fear of fat	3.60 (2.71)	4.61 (2.51)	5.18 (2.31)	5.35 (2.66)	*F*_(3)_ = 13.212	NW > UW*	−0.40 (−0.65 to −0.14)
						OW > UW***	−0.66 (−0.93 to −0.39)
						O > UW***	−0.65 (−0.92 to −0.39)
						OW > NW**	−0.23 (−0.37 to −0.09)
						O > NW***	−0.29 (−0.43 to −0.15)
Willpower	4.19 (2.20)	4.15 (2.32)	3.80 (2.14)	3.17 (2.05)	*F*_(3)_ = 14.395	UW > O**	0.49 (0.22 to 0.76)
						NW > O***	0.44 (0.30 to 0.58)
						OW > O**	0.30 (0.14 to 0.46)

Abbreviations: NW, normal weight; O, obesity; OW, overweight; SD, standard deviation; UW, underweight.

* *p* < .05;

** *p* < .01;

*** *p* < .001.

In relation to *Fear of Fat* (*F* = 13.212, df = 3, *p* < .01), there was also a significant difference across BMI groups. Overweight participants reported suffering more fear of gaining weight than underweight participants (*p* < .001, Hedges' *g* = −0.66, 95% CI = −0.39 to −0.93) and normal weight participants (*p* < .01, Hedges' *g* = −0.23, 95% CI = −0.09 to −0.37). However, participants with obesity presented more fear than underweight participants (*p* < .001, Hedges' *g* = −0.65, 95% CI = −0.39 to −0.92) and normal weight people (*p* < .001, Hedges' *g* = −0.29, 95% CI = −0.15 to −0.43). Participants with normal weight also present more fear of gaining weight compared to underweight participants (*p* < .05, Hedges' *g* = −0.40, 95% CI = −0.14 to −0.65). Non statistically significant difference was found between participants with overweight and with obesity in terms of fear of gaining weight.

Finally, for *Lack of willpower* (*F* = 14.395, df = 3, *p* < .01), only participants with obesity were those who were less convinced that obesity is under an individual's control. Underweight participants (*p* < .01, Hedges' *g* = 0.49, 95% CI = 0.22 to 0.76), normal weight (*p* < .001, Hedges' *g* = 0.44, 95% CI = 0.30 to 0.58) and overweight participants (*p* < .001, Hedges' *g* = 0.30, 95% CI = 0.14 to 0.46) considered that obesity was caused by a lack of willpower, compared to participants with obesity.

Comparisons were also conducted between men and women (see Table 6). Compared to men, women reported feeling more fear of gaining weight (*p* < .001, Hedges' *g* = 0.30, 95% CI = 0.16 to 0.43). However, men presented more negative attitudes and beliefs towards obesity (*p* < .001, Hedges' *g* = −0.26, 95% CI = −0.13 to −0.40) and were also more likely to believe that obesity is a result of a lack of willpower and is under an individual's control (*p* < .001, Hedges' *g* = −0.4, 95% CI = −0.28 to −0.55).

**TABLE 6:** Comparisons of the AFA among sex

	Women mean (SD)	Men mean (SD)	Student's *t*	Hedges' *g* (95% CI)
Dislike	2.16 (1.17)	2.49 (1.54)	*t*_(371.650)_ = 3.330**	−0.26 (−0.40 to −0.13)
Fear of fat	5.06 (2.56)	4.31 (2.42)	*t*_(1246)_ = −4.350***	0.30 (0.16 to 0.43)
Willpower	3.61 (2.19)	4.52 (2.26)	*t*_(1246)_ = 6.100***	−0.41 (−0.55 to −0.28)

* *p* < .05; **p < .01;

*** *p* < .001.

There were also differences between levels of income. *Dislike* differed in the different categories of income (*F* = 4.181, df = 3, *p* < .01). Specifically, participants with incomes lower than 2.5 times the minimum wage (MW) reported more negative attitudes towards obesity than those with incomes below the MW (*p* < .05, Hedges' *g* = −0.30, 95% CI = −0.11 to −0.49). There was also a difference in beliefs about *Willpower* (*F* = 3.881, df = 3, *p* < .01). Participants with higher incomes seemed to believe that obesity is under everybody's control. Participants with incomes below 2.5 times the MW (*p* < .05, Hedges' *g* = −0.30, 95% CI = −0.10 to −0.50) and incomes below 2 times the MW (*p* < .05, Hedges' *g* = −0.24, 95% CI = −0.08 to −0.40) were more convinced that obesity is caused by a lack of willpower than participants with no incomes.

Regarding education level, there were significant differences between groups in the *Dislike* subscale (*F* = 3.598, df = 2, *p* < .05). Participants who had completed higher education had more negative attitudes towards obesity than participants who had only completed secondary education (*p* < .05, Hedges' *g* = −0.16, 95% CI = −0.04 to −0.28).

Correlation analyses revealed that the people with the highest BMI suffered the most fear of gaining weight (*r* = .152, *p* < .001). However, on the contrary, they had fewer negative attitudes towards obesity (*r* = −.113, *p* < .001) and were more conscious that obesity is not under an individual's control (*r* = −.182, p < .001). As Table 4 shows, there was a strong positive correlation between subtle and blatant discrimination (*r* = .720, *p* < .001). These results predict that those who perceive conscious discriminative behaviours also perceive unconscious discriminative ones. There was also a significant correlation between *Dislike* and *Willpower*, indicating that people who considered obesity as something controllable presented more negative attitudes towards obesity (*r* = .403, *p* < .001).

## 4 DISCUSSION

The aims of the present study were threefold. The first goal was to assess the internal structure of the scale using a PCA and a CFA. Second, analyse the internal consistency and conduct a test–retest of the scale. The third goal was to study whether there were differences in anti‐fat attitudes among sex, BMI categories and other sociodemographic variables. As a result, this is the first study to assess the psychometric properties of the scale in a large Spanish community sample, including all BMI categories, taking into consideration that people with overweight and obesity were excluded in the original Spanish adaptation of the AFA.^37^ At the same time, it is the first research that study 1 month test–retest reliability of the scale and the first to assess differences in anti‐fat attitudes among income and educational level in a Spanish sample. We hope these new contributions will fill some gaps and add more relevant information in the field of weight discrimination.

In order to achieve the first goal of the study, a cross‐validation was applied to analyse the internal structure of the scale. The general sample was then divided into two independent samples in order to conduct the PCA and CFA respectively. In general, the psychometric properties of the scale were adequate. The PCA revealed the existence of three factors, and the CFA confirmed this structure since the data showed a good fit to the proposed model, according to Hu and Bentler.^43^

It should be noted that the lowest factor loading was found on item 2, which assesses whether the person has friends or acquaintances who are overweight or have obesity. Similar results were found in the former Spanish validation with college students,^47^ but this is something that is not present in the original scale.^36^ However, the factor loading of this item indicated the adequacy of its inclusion in the questionnaire according to Akoglu^45^ and it was therefore retained in the scale to assess negative attitudes towards obesity. Overall, the PCA and CFA corroborated the multi‐factorial structure of the scale. This was consistent with previous literature.^36^, ^37^

The reliability of the study showed adequate Cronbach's alpha and McDonald's omega values, according to Bland and Altman.^44^ Additionally, reliability was also assessed using the test–retest method, this being the first study to assess the 1‐month stability of anti‐fat attitudes. Correlations of scores obtained over time were strong among the three factors of the scale.^45^

The convergent validity of the scale was adequate in terms of correlations between the AFA subscales and the MSPD scale which measures blatant and subtle discrimination. The results reinforced the strong correlation between implicit and explicit discrimination related to weight discrimination. Moreover, the results also showed that attributions of behavioural causes of obesity were linked to negative attitudes towards obesity. This highlighted what weight discrimination is based on, as people believe obesity is something related to food intake and physical activity,^48^ aspects that everyone is supposed to be able to control. As similar research revealed previously, attributions of behavioural causes of obesity were the predictor of stronger weight bias, even across four countries so different in sociocultural factors such as Canada, the United States, Iceland and Australia.^48^ This data from our study reinforced the importance of working on the reduction of the stereotypes associated with obesity as a possible target to reduce weight discrimination. A significant positive correlation also stressed out that people with more dislike towards people with obesity presented more fear of gaining weight. Despite the weak correlation, it would be interesting to study this relationship using a sample of people with strong control weight behaviours, such as anorexia nervosa or other feeding or eating disorders, while including this aspect in clinical settings.

Additionally, the relationship between AFA scores and several sociodemographic variables was analysed. We expected that men would reveal more dislike towards obesity, but that women would report more fear of gaining weight. Moreover, it was also expected to find high dislike towards people with obesity also in participants with overweight or obesity.

In general, normal weight participants showed more dislike towards people with obesity than other BMI groups. Participants with overweight and obesity presented the lowest anti‐fat attitudes. The results were statistically significant and rejected the idea that there is intra‐group discrimination between people with overweight or obesity, contrary to what was expected in our initial research hypothesis. Despite some previous studies presented similar results,^49^ there is some discrepancy in this regard, as other studies found that people with overweight and obesity also present negative attitudes about obesity, both implicit and explicit ones.^50^, ^51^, ^52^ The results were also divergent when assessing by individual's or nation's dislike towards obesity. A study across 71 countries revealed that higher BMI scores were associated with lower dislike towards overweight (both implicit and explicit dislike) at an individual level, but not when controlling for overweight at a national level (i.e., nations with higher rates of overweight).^53^ It evinces that further research would be necessary to reject or confirm this idea, and to study what are the implied mechanisms (e.g., empathy) that explain why people with obesity also present negative attitudes towards other individuals with obesity. However, what is clear is that all the results reinforced the idea of a preference for thin people.

The present study also revealed that underweight, normal weight and overweight participants believed that obesity is something controllable and influenced by behavioural aspects, while participants with obesity were more convinced that obesity is not only attributable to behavioural facets. It may imply that this perception could be influenced by their knowledge about obesity or due to their own experiences. But, at the same time, it is striking that this perception was not present in the overweight participants.

The results also revealed that people with overweight and obesity endured more fear of gaining weight than underweight and normal weight participants and are in line with previous studies.^49^ This result can be explained by being previously discriminated against weight reasons, but it was not controlled in the present study. As mentioned earlier, people with a higher BMI endure more weight discriminating experiences, and therefore their fear could be logical if they want to stop being discriminated against due to their weight. However, more research and special longitudinal studies are needed.

The results of the current study also revealed that women reported fewer negative attitudes towards obesity and that they were more conscious that obesity is not attributable to behavioural characteristics. This is consistent with previous studies that found less weight discrimination in women than men.^48^, ^54^ However, as seen in previous studies^54^ women reported more *Fear of Fat*, which means that they were more worried about weight. These results are similar to those of others studies^49^ and confirmed our initial hypotheses. Additionally, it seems to be another signal of the undoubtedly pressure that women face in our society in terms of body weight. Consequently, it can drive to important repercussions. For example, some previous studies have already found negative relations between depicting thin‐ideal body exposure in mass media and harmful consequences such as body dissatisfaction or internalization of the thin ideal in women.^55^

Data from the present sample suggests that income and educational level are also related to negative attitudes towards obesity. Specifically, people with incomes greater than 2.5 times the minimum wage and people who had completed higher education presented more weight bias. These results are consistent with other studies and especially with a systematic review that indicated that weight discrimination attitudes increase with higher education and higher income.^56^

Overall, the evidence indicates that the people who are most likely to present weight discrimination are normal weight men, with an income greater than 2.5 times the minimum wage and well qualified. These indicators suggest that reducing weight bias interventions might put emphasis on this target population. Despite that, more research would be necessary to confirm it.

This study used a large Spanish sample that came from the Internet and included people among all BMI categories from underweight to obesity. Hence, considering the access of numerous people to the Internet and the representation of participants with the different BMI categories, we believe it could be a representative sample of the general Spanish population. According to constraints on generality (COG) guidelines,^57^ given the sample size and that the results were in concordance with previous studies, we expect our findings to be generalized to other populations with similar sociodemographic characteristics (including all BMI ranges) and cultural characteristics. Also given the sample size and that the sample came from the general population, we predict this validation can be used to assess anti‐fat attitudes in other Spanish language countries. Regarding replication, as the study did not have any particularities to be conducted, we predict it is easy to replicate and we encourage researchers to replicate it using similar or different sociocultural samples.

The present study had some strengths and added new data regarding anti‐fat attitudes, specially using a different sample apart from the most common research conducted with samples from the United States. In this way, the present study reflects and adds different language and cultural aspects to the field of weight discrimination. Despite that, further research using samples from different languages and cultural backgrounds could be interesting.

However, some limitations should also be mentioned. Even though the size of the sample was big, men were underrepresented. This is a common feature of studies focused on weight or eating behaviours. A challenge for future research will be engaging more men to participate in these types of studies. A second limitation is related to how the income variable was measured. In the present study, a categorical item was used, which provided an ordinal scale for income, but a continuous measure would provide more accurate information about this variable. Further research is also needed when studying the change in negative attitudes towards obesity in participants that were overweight or had obesity in the past and are now normal weight. It would be interesting to explore how attitudes and beliefs about obesity possibly change according to a participant's past discrimination.

In conclusion, the present study shows adequate psychometric properties of the AFA in a sample of the Spanish general population, including participants with overweight and obesity. This validation could have some different practical implications as it can be used in assessing negative attitudes towards obesity, studying mechanisms that explain weight discrimination and its maintenance, designing preventive programs to reduce weight bias and/or conducting further research on anti‐fat attitudes using different samples. Therefore, this scale is a good instrument for assessing the weight discrimination construct in research and clinical settings.

## AUTHOR CONTRIBUTIONS

Sergio Macho was in charge of collecting all the data and carrying out all the statistical analyses. Ana Andrés and Carmina Saldaña oversaw all the study. All the authors were involved in the writing of the paper.

## CONFLICT OF INTEREST

No conflict of interest was declared.
